# Loop-mediated isothermal amplification (LAMP) approach for detection of heat-resistant *Talaromyces flavus* species

**DOI:** 10.1038/s41598-019-42275-x

**Published:** 2019-04-10

**Authors:** Jacek Panek, Magdalena Frąc

**Affiliations:** Institute of Agrophysics, Polish Academy of Sciences, Doświadczalna 4, 20-290 Lublin, Poland

## Abstract

*Talaromyces flavus* is a soilborne fungus that can contaminate fruits. It constitutes serious influence on heat-processed food spoilage, as *T. flavus* belongs to the heat-resistant fungi group, which are able to survive the pasteurization process. Moreover *T. flavus* has been reported to be capable of mycotoxigenicity, therefore they have a serious threat to human health. To maintain the safety of food production, sensitive method for *T. flavus* detection was developed. The loop mediated amplification, abbreviated LAMP, reactions were designed as specific for detection of *DNA replication licensing factor* gene of *T. flavus*. The specificity of assay was confirmed by use of 5 *T. flavus* strains and 35 other fungal isolates. The achieved limit of detection was 1fg of *T. flavus* genomic DNA and 64 ascospores in 1 g of strawberry fruits or soil samples.

## Introduction

Fungal spoilage of heat-processed food emerges as a major problem in food industry. Estimated losses caused to fruit juice production by heat-resistant fungi (HRF) cost food industry millions of USD solely in United States of America^1,2^. HRF were reported to inhabit soil and contaminate agricultural products in countries around the world, making them a worldwide threat^1,3–8^. Aforementioned spoilage origins from HRF that contaminate raw agricultural products by either anemochory or physical contact with soil that is inhabited by HRF such as *Talaromyces flavus*^3,5,9^.

*T. flavus* is one of the most common fungi among HRF group. The HRF are able to withstand high temperature treatment, such as pasteurization process^10,11^. Heat resistance of *T. flavus* labelled as decimal reduction time (D-value) was determined to achieve D_90_ °C = 6 minutes and D_95_ °C = 1 minute^12^. Furthermore, *T. flavus* was reported to possess capability of producing plenty of mycotoxins and to grow under microaerobic and aerobic conditions^13–16^. What is also significant, other HRF *Neosartorya fischeri* was reported to be resistant to such growth control factors as fungicides or antibiotics^17^. These findings and these traits further establish *T. flavus* and HRF as a potent risk factor to the food industry and a risk for consumer health. Moreover, currently the best remedy to provide HRF-free heat-processed products is detection of the HRF in raw products or in soil under plantations. To achieve this, successful methods based on PCR and qPCR techniques to detect HRF of genus *Neosartorya, Byssochlamys* and species *Talaromyces flavus* were developed^18–20^. Yet, emergence of isothermal amplification techniques may provide the possibility to develop HRF detection methods that can be regarded as easier and cheaper assay to deploy within food industry and on the field. Among the isothermal amplification methods, the ones that are used the most commonly, due to necessity to use only one enzyme, are Rolling circle amplification (RCA), Cross-priming amplification (CPA), Polymerase spiral reaction (PSR) and Loop-mediated isothermal amplification (LAMP)^21–24^.

The aim of this study was to develop LAMP method for detection of *Talaromyces flavus* that provides specificity and rapidity of achieving results. LAMP is a technique that due to use of 6 primers that are complementary to 8 regions of DNA sequence assures very high specificity^24,25^. Moreover, the isothermal character of reaction and presence of many priming sites for DNA synthesis provides possibility to observe results earlier than in competing techniques such as PCR or qPCR. Apart from that, LAMP reaction provides possibility to detect occurrence of amplification product by either measurement fluorescence of minor-groove dsDNA binding dyes, by measurement of increasing turbidity of reaction or by colorimetric detection using pH sensitive dyes. It is important to mention, that described detection methods can render the results visible to the naked eye. Moreover, laboratory equipment such as qPCR thermocyclers, fluorimeters or turbidimeters may be used to visualise the results^26–31^. The target of developed reaction was *Talaromyces flavus DNA replication licensing factor (rlf)* gene. The DNA licensing factor is a protein complex that allows replication to begin at the replication origin site and is a part of the replication licensing system, which regulates the replication and ensures that no section of genome is replicated more than once in a single cell cycle^32^. The *DNA replication licensing factor* has already been reported as a possible marker and target gene for detection of *T. flavus* by qPCR reaction^20^.

## Material and Methods

### Development of the LAMP method

**Figure 1:**
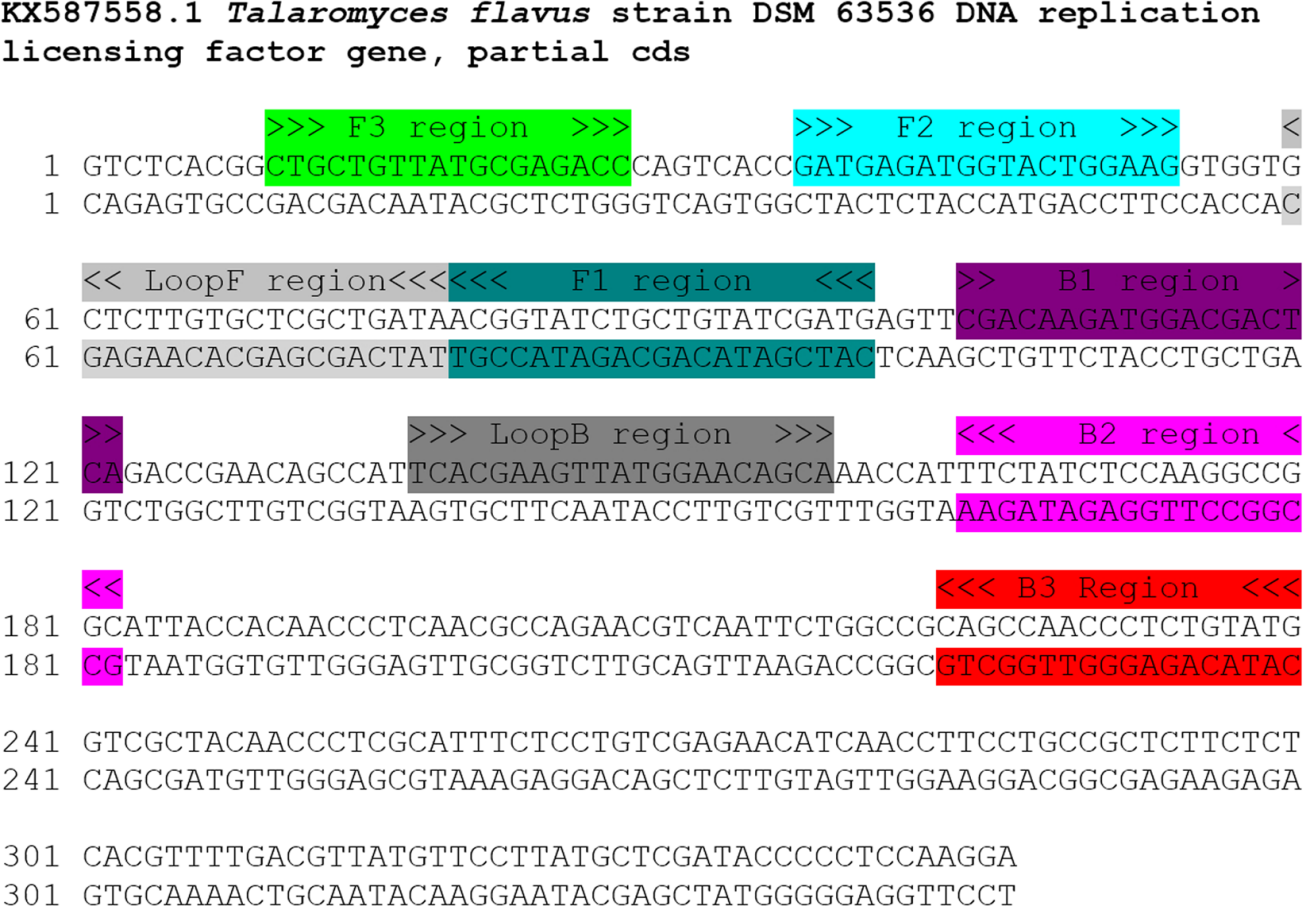
Positioning and orientation of regions used to design LAMP primers within the nucleotide sequence of the DNA replication licensing factor gene of *T. flavus* DSM 63536 (GenBank accession no. KX587558).

In order to design LAMP primers, 10 nucleotide sequences of *T. flavus rlf gene* (accession number GU396448.1 - GU396452.1, KX587558-KX587562) were obtained from GenBank database and aligned with MEGA6 software^33^. After that, possible regions for primer design were found and checked by BLAST performed within GenBank database to ensure the uniquity and specificity of them^34^. Then, the primers were designed with help of LAMP Designer software (Optigene) and once again confirmed by BLAST to reassure their specificity (Table 1 and Fig. 1). During the assessment of specificity of the designed LAMP reaction, DNA extracted from 40 fungi isolates (Table 2) was used. Employed strains were identified prior by sequencing of internal transcribed spacer 1 (ITS1) and/or D2 region of large subunit of rRNA gene (D2 LSU rRNA), with comparison to MicroSeq, GenBank and Mycobank databases. The studied pool of microorganisms consisted of 5 *T. flavus* strains commercially available from Leibniz Institute DSMZ-German Collection of Microorganisms and Cell Cultures (Braunschweig, Germany) and from NITE Biological Resorce Center (Tokyo, Japan), and collection of 35 different isolates containing other HRF, cosmopolite fungi and fungi isolated from soil and strawberries belonging to genus *Talaromyces, Penicillium, Aspergillus, Neosartorya* and *Byssochlamys*.

**Table 1:** Primers for the LAMP assay.

Species	Targeted sequence	Primer name	Primer sequences 5′→3′	Final concentration
*Talaromyces flavus*	DNA replication licensing factor gene	Tf_F3	CTGCTGTTATGCGAGACC	0.25 µM
		Tf_B3	CATACAGAGGGTTGGCTG	0.25 µM
		Tf_FIP	CATCGATRCAGCAGATACCGTGATGAGATGGTRCTGGAAG	0.5 µM
		Tf_BIP	CGACAAGATGGACGACTCRGCCGGCCTTGGAGATAGAA	0.25 µM
		Tf_LoopF	TATCAGCGAGCACAAGASC	0.5 µM
		Tf_LoopB	TCACGAAGTTATGGAACAGCA	0.25 µM

**Table 2:** List of fungal strains used and LAMP specificity results.

Fungi	Reference	Isolation source	Accession number of sequences in GenBank (^a^RLF, ^b^D2 LSU, ^c^ITS1)	LAMP results
*Talaromyces flavus*	DSM 63536	Nepal; soil	^a^KX587558, ^b^KX587552, ^c^MG878427	+
*Talaromyces flavus*	NBRC 31879	Unknown	^a^KX587559, ^b^KX587553, ^c^MG878428	+
*Talaromyces flavus*	NBRC 30574	Nepal; soil	^a^KX587560, ^b^KX587554, ^c^MG878429	+
*Talaromyces flavus*	NBRC 7233	Japan; unknown	^a^KX587561, ^b^KX587555, ^c^MG878430	+
*Talaromyces flavus*	NBRC 102293	Japan; grassland	^a^KX587562, ^b^KX587556, ^c^MG878431	+
*Talaromyces macrosporus*	NBRC 30070	Unknown; photo album	^b^KX587557, ^c^MG878432	−
*Byssochlamys nivea*	LMEM G49/11	Poland; fruit-based silage	^b^MG877737	−
*Neosartorya fischeri*	LMEM G48/12	Poland; strawberry jam	^b^KC179765	−
*Byssochlamys fulva*	DSM 62097	Germany; bottled apple juice	^b^MG877729	−
*Byssochlamys fulva*	DSM 1808	Unknown; grape juice	^b^MG877730	−
*Neosartorya glabra*	LMEM G5/14	Poland; soil	^b^MG877735	−
*Talaromyces ruber*	LMEM G141/14	Poland; soil	^b^KM822632	−
*Talaromyces islandicus*	LMEM G142/14	Poland; soil	^b^KM822633	−
*Penicillium camembertii*	LMEM G77/11	Poland; grass silage	^b^MG877736	−
*Aspergillus niger*	LMEM G99/13	Poland; soil	^b^MG877738	−
*Aspergillus niger*	LMEM G100/13	Poland; soil	^b^MG877739	−
*Aspergillus niger*	LMEM G101/13	Poland; soil	^b^MG877740	−
*Aspergillus niger*	LMEM G102/13	Poland; soil	^b^MG877741	−
*Aspergillus niger*	LMEM G105/13	Poland; soil	^b^MG877742	−
*Penicillium miczynskii*	LMEM G4/18	Poland; soil	^b^MG877731	−
*Talaromyces angelicus*	LMEM G5/18	Poland; soil	^b^MG877732	−
*Aspergillus fumigatus*	LMEM G6/18	Poland; soil	^b^MG877733	−
*Talaromyces angelicus*	LMEM G7/18	Poland; strawberries	^b^MG877734	−
*Talaromyces angelicus*	LMEM G8/18	Poland; strawberries	^b^MG881801, ^c^MG878420	−
*Talaromyces angelicus*	LMEM G9/18	Poland; strawberries	^b^MG881802, ^c^MG878421	−
*Talaromyces kabodanensis*	LMEM G10/18	Poland; strawberries	^b^MG881803, ^c^MG878422	−
*Talaromyces kabodanensis*	LMEM G11/18	Poland; strawberries	^b^MG881804, ^c^MG878423	−
*Talaromyces kabodanensis*	LMEM G12/18	Poland; strawberries	^b^MG881805, ^c^MG878424	−
*Talaromyces kabodanensis*	LMEM G13/18	Poland; strawberries	^b^MG881806, ^c^MG878425	−
*Talaromyces aculeatus*	LMEM G14/18	Poland; strawberries	^b^MG881807, ^c^MG878426	−
*Talaromyces kabodanensis*	LMEM G15/18	Poland; soil	^b^MG881808, ^c^MG878410	−
*Talaromyces kabodanensis*	LMEM G16/18	Poland; soil	^b^MG881809, ^c^MG878411	−
*Talaromyces kabodanensis*	LMEM G17/18	Poland; soil	^b^MG881810, ^c^MG878412	−
*Talaromyces kabodanensis*	LMEM G18/18	Poland; soil	^b^MG881811, ^c^MG878413	−
*Talaromyces kabodanensis*	LMEM G19/18	Poland; soil	^b^MG881812, ^c^MG878414	−
*Talaromyces kabodanensis*	LMEM G20/18	Poland; soil	^b^MG881813, ^c^MG878415	−
*Talaromyces kabodanensis*	LMEM G21/18	Poland; soil	^b^MG881814, ^c^MG878416	−
*Talaromyces kabodanensis*	LMEM G22/18	Poland; soil	^b^MG881815, ^c^MG878417	−
*Talaromyces kabodanensis*	LMEM G23/18	Poland; soil	^b^MG881816, ^c^MG878418	−
*Talaromyces kabodanensis*	LMEM G24/18	Poland; soil	^b^MG881817, ^c^MG878419	−

DSM – Leibniz Institute DSMZ-German Collection of Microorganisms and Cell Cultures, Braunschweig, Germany; NBRC – National Institute of Technology and Evaluation, Tokyo, Japan; LMEM – Laboratory of Molecular and Environmental Microbiology, Institute of Agrophysics, Polish Academy of Sciences, Lublin, Poland.

The tested fungal species were grown on Potato Dextrose Agar (PDA) at 27 °C for 7 days. The DNA isolation and quality assessment was performed as in previous work^20^. The mycelium was homogenized in sterile conditions with using of FastPrep-24 instrument (MP Biomedicals) under the following settings: 6 m/s, 40 seconds, and with the matrix containing glass beads with diameter 3.15 mm (500 mg) and 1.45 mm (250 mg), and reagents: 400 µl of Lyse F buffer (EURx), 10 µl of Proteinase K (EURx) and 3 µl of RNase A (EURx). Next, the mycelium was sonicated in 240 W ultrasonic cleaner (Polsonic SONIC-6) for 10 minutes, After disintegration the fungal genomic DNA was extracted according to manufacturer protocol with Plant & Fungi DNA Purification Kit (EURx) and suspended in 200 µl of Tris-HCl buffer (10 mM Tris-HCl, pH 8.5). The quality and quantity of the DNA was determined spectrophotometrically by NanoDrop instrument (Thermo Fisher Scientific) and fluorometrically with Quantus instrument (Promega) and deemed as good for further analysis (A_260_/A_280_ = 2.05 SD = 0.08 while A_260_/A_230_ = 1.94 SD = 0.06).

In order to determine if developed method can be used with quick DNA extraction methods, PrepMan™ Ultra Sample Preparation Reagent (Thermo Fisher Scientific) was used. The mycelium of tested fungal species, and also soil samples or strawberry fruits were transferred with using 10 µl inoculation loop into 1.5 ml Eppendorf tube containing 100 µl of PrepMan reagent, thoroughly vortexed and heated to 100 °C for 10 minutes. After that, samples were cooled to the room temperature for 2 minutes and centrifuged (120 s, 20 000 xg). 50 µl of supernatant was preserved (−20 °C) for reactions.

The LAMP reactions were performed with 7500 Fast thermocycler (Applied Biosystems). The reaction contained 6 μl of Isothermal MasterMix (ISO-001, OptiGene), 3 μl of PrimerMix (Tables 1) and 1 μl of the DNA sample. Total reaction volume was 10 μl. Reactions were conducted in constant temperature of 65 °C through 90 min with fluorescence data collection every 1 minute. Isothermal MasterMix contained GspSSD polymerase with strand displacement activity and fluorescent dye intercalating to dsDNA minor-groove compatible with FAM channel. To determine the detection limit of developed reactions serial 10-fold dilutions of *T. flavus* DSM 63536 genomic DNA solution obtained with EURx method (100 ng/μl) and PrepMan method (0.1 ng/μl) were prepared. Concentrations of 10 ng/µl, 1 ng/µl, 100 pg/µl, 10 pg/µl, 1 pg/µl, 100 fg/µl, 10 fg/µl and 1 fg/µl (EURx method) and 100 pg/µl, 10 pg/µl, 1 pg/µl, 100 fg/µl, 10 fg/µl and 1 fg/µl (PrepMan method) were obtained and 1 µl of each was used as a template for LAMP reaction. Sterilized DirectQ water was used as non-template control (NTC). All reactions were performed in biological triplicates. The results were visualized as amplification plots (∆R) expressed as raw fluorescence units (RFU) in time. To obtain ∆R, on the basis of the raw fluorescence values, a baseline was subtracted from the raw data. Presence or absence of amplification plot determined the positivity or negativity of tested reactions. Moreover, cycle thresholds were calculated with assumption that each data collection step corresponds to 1 cycle. Each reaction was followed with melting curve analysis (65 °C to 95 °C, ∆0.016 °C/s).

### Application of LAMP method using environmental samples

To determine applicability of developed LAMP method for detection of *T. flavus* in food and soil samples the LAMP assays were performed with DNA isolated from artificially contaminated environmental samples - strawberry fruits and soil, which is a source of fungal contamination of agricultural products and food.

In this study, soil samples, which were obtained from strawberry plantations in Poland, Lubelskie voivodeship and strawberries fruits of *Fragaria* × *ananassa* ‘Senga Sengana’ cultivar were contaminated with ascospores of *T. flavus*. To confirm absence of natural occurrence of *T. flavus* in examined environmental samples the method of Beuchat and Pitt^4^ was used for fungal strains isolation. Achieved isolates were additionally analysed and identified by sequencing of D2LSU rRNA gene. The results confirmed, that studied samples were free of natural occurrence of *T. flavus*. Environmental samples were not sterilised prior to study to ensure, that designed method is able to detect *T. flavus* DNA in presence of other, autochthonic DNA. *T. flavus* DSM 63536 ascospores were suspended in sterile water and vortexed for 30 seconds. The concentration of suspension was measured microscopically with Thoma’s counting chamber and resulted in 64000 ascospores per 1 ml of sterile water. Then, serial 10-fold dilutions (64000, 6400, 640 and 64 ascospores per 1 ml of water) of suspension were prepared. After that, 1 ml of each prepared inoculum was added to 1 g of strawberry fruits and soil samples and then, mixtures were vortexed for 15 seconds. Next, 200 ml of contaminated sample was transferred to sterile tubes. NTC samples were prepared by addition of sterile water to soil and strawberry fruits samples instead of inoculum. To isolate DNA from contaminated strawberry fruits, already described above procedure involving EURx Plant & Fungi DNA Purification kit and also PrepMan Ultra Sample Preparation Reagent were used. Extraction from contaminated soil samples was conducted with Soil DNA Purification Kit (EURx) and PrepMan Ultra Sample Preparation Reagent according to manufacturer’s protocols.

### Validation of developed method

In order to further determine the potential applicability of developed approach, validation tests were performed on strawberry fruits and soil samples obtained from strawberry plantations located in Lublin Voivodeship, Poland. Samples were prior examined with use of standard method for detection of HRF^4^ with addition of sequencing of D2LSU rRNA gene. DNA was extracted from 10 soil and 10 strawberry fruits samples with above mentioned EURx and PrepMan methods.

## Results

### Specificity and sensitivity of LAMP method

**Figure 2:**
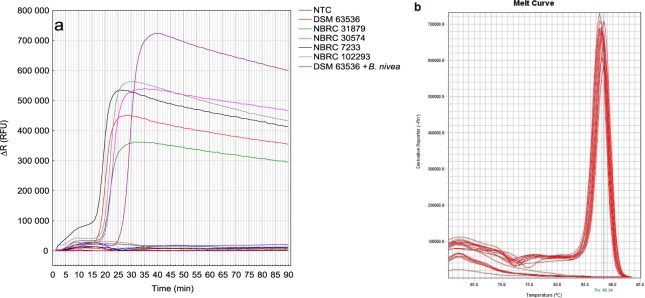
The specificity of *Talaromyces flavus* LAMP detection method. (**a**) The amplification plots (∆R) of *T. flavus* and 35 other studied isolates (Table 2) in time. Legend describes tested *T. flavus* isolates, while rest of tested strains are described in Table 2. NTC stands for non-template control. (**b**) Melting curve analysis of obtained products. DNA isolated with EURx Plant&Fungi DNA Purification Kit.

Developed LAMP reaction for detection of *T. flavus* provided specific results. Positive amplification results were observed with all five *T. flavus* isolates: *T. flavus* DSM 63536, *T. flavus* NBRC 31879, *T. flavus* NBRC 30574, *T. flavus* NBRC 7233 and *T. flavus* NBRC 102293, and with *T. flavus* DSM 63536 DNA mixed with *Byssochlamys nivea* LMEM G49/11 DNA in ratio of 100fg:10 ng of genomic DNA, while no amplification was observed with DNA templates isolated from other tested 35 fungal isolates (Fig. 2a). Calculated Ct of positive reactions was 19.47, SD ± 2.72. Figure 2b presents melting curves of obtained amplification products. The fact that proposed reaction did not amplify DNA isolated from fungi commonly found in environmental samples belonging to genus *Penicillium* and *Aspergillus*, nor from other common HRF like *Byssochlamys, Neosartorya* and other species of *Talaromyces* sp., isolated from soil or strawberry fruits samples, suggest that developed reaction is very specific. What is more, the same results were obtained for fungal DNA isolated with PrepMan extraction method. Positive reaction observed in samples containing mixed DNA of *T. flavus* and *B. nivea* in ratio of 1:100000 suggest, that presence of external DNA does not interfere with amplification.

### Sensitivity of LAMP method for detection of *T. flavus*

**Figure 3:**
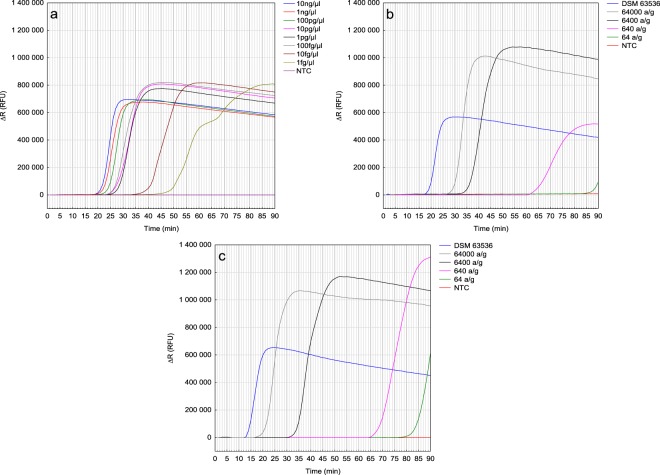
Sensitivity study of *Talaromyces flavus* LAMP detection method. (**a**) The amplification plots (∆R) of pure genomic *T. flavus* DSM 63536 DNA added into reaction in different concentrations in time. (**b**) The amplification plot (∆R) of *T. flavus* detected in strawberries contaminated by different number of ascospores added to 1 g of sample (a/g) prior the DNA isolation reaction. DNA isolated with EURx Plant&Fungi DNA Purification Kit. (**c**) The amplification plot (∆R) of *T. flavus* detected in soil contaminated by different number of ascospores added to 1 g of sample prior the DNA isolation reaction. NTC stands for non-template control. DNA isolated with EURx Soil DNA Purification Kit.

The amplification plots visible at the Fig. 3 represent the results of LAMP sensitivity assay. In order to assess the sensitivity of developed LAMP approach, reactions were prepared with serially diluted DNA matrices. With use of EURx DNA extraction method, positive results were observed for all tested DNA matrix concentrations ranging from 10 ng/µl to 1 fg/µl, yet no amplification was observed in non-template controls (Fig. 3a). Obtained results show that the limit of detection for proposed reaction is 1 fg/µl. Results for DNA matrix concentrations of 10 ng/µl to 100 fg/µl were observed in about 30 minutes (Ct of 23.29, SD ± 2.97), while positive results of reactions with DNA matrices of 10 fg/µl and 1 fg/µl were observed after about 45 (Ct of 37.42, SD ± 1.29) and 55 minutes (Ct of 44.68, SD ± 4.62), respectively. With deployment of PrepMan DNA extraction method, the positive results were observed in range of 100 pg/µl to 10 fg/µl in all replications (Ct 23.03, SD ± 1.35; Ct 35.99, SD ± 1.81; Ct 38.6, SD ± 2.46; Ct 55.27, SD ± 3.48; Ct 71.2, SD ± 3.52). As for matrix containing 1 fg/µl, weak positive results were observed in only one repetition (Ct 88.72).

The sensitivity assessments of *T. flavus* detection from contaminated strawberries and soil are presented at Fig. 3b,c, respectively. LAMP reactions resulted in positive amplifications plots. The assay was able to detect occurrence of DNA isolated with EURx methods from samples inoculated with 64 ascospores per 1 g of each tested environmental sample types: soil and strawberry fruits. Time needed to observe positive results was similar for both kinds of samples, and resulted in about 90 minutes, about 75 minutes, about 40 minutes and about 30 minutes for DNA isolated from 64, 640, 6400 and 64000 ascospores per 1 gram of sample, respectively. Simultaneously no amplification was observed in NTC. These results indicate that the developed LAMP method can accurately detect *T. flavus* ascospores in strawberry fruits and soil samples. Moreover, developed assay was able to positively amplify DNA isolated with PrepMan method from soil samples contaminated with 640 or more ascospores per 1 g. However, in case of DNA extracted with the same method from contaminated strawberry fruits, the detection was possible only in the highest concentration of ascospores (64 000 per 1 g).

### Validation of developed method

Out of 10 tested soil samples 4 were prior identified as spoiled with *T. flavus*. Both tested method of DNA extraction allowed to detect *T. flavus* in those samples, however with EURx method, one more sample was positive. In case of strawberry fruits samples, out of 5 samples identified prior as contaminated with *T. flavus*, positive reaction results were observed in all of these 5 samples, when DNA was extracted with EURx method and only in one sample extracted with PrepMan method.

## Discussion

Studies towards identification and preservation of fungal contamination in food products are important to reassure high quality and health safety of food products. Heat-resistant fungi play important role in endangering food production as they are able to survive the pasteurization process. Moreover, as they in general are not considered plant pathogens, symptoms of their occurrence on fruit may not be observed. These attributes give rise to claim that one of the best methods to provide HRF-free food production and safety involves early detection of HRF contamination in raw products^9^. It is important, that food processing industry often requires quality control methods that are rapid, sensitive and specific. The common, traditional agar plate based microbiological methods do not provide, especially in case of quickness and specificity^35^. What is more, the other methods, such as liquid chromatography-mass spectrometry methods developed in recent years can determine mycotoxins to evaluate postharvest quality of fruits^36^ by they don’t detect the presence of fungi at the very early stage before contamination of samples by toxins. It is important to say, that presence of HRF contamination, even without mycotoxin secretion, presents threat to the food production, as it may spoil the final product during its shelf life. To fulfil these requirements, molecular biology techniques were vastly employed, especially to detect bacteria and fungi causing human diseases, yet the HRF occurrence detection seem to be neglected^37,38^. In recent years, PCR and qPCR based methods for detection of HRF genus *Neosartorya*^19^, genus *Thermoascus*^38^, genus *Byssochalmys* and *Hamigera*^18^ and species *Talaromyces flavus*^20^. Due to emergence of isothermal techniques such as LAMP, researchers developed methods to utilize these techniques to detect microorganisms and viruses important for the animal and plant food processing and consumer health^30,39–44^ and in general public health *sensu largo*^45–51^. However, LAMP methods for detection of fungi are scarce, while there are no isothermal methods concentrated on detecting any of HRF, yet alone *Talaromyces flavus*.

In this study a loop-mediated isothermal amplification method for detection of *T. flavus* was developed. The developed assay was evaluated for detection of *T. flavus* in environmental samples, such as strawberry fruits and soil samples. Designed method and primers were confirmed to be specific and to discriminate *T. flavus* from other *Talaromyces* species, HRF and common cosmopolite fungi. Designed method targeted *Talaromyces flavus DNA replication licensing factor gene*, which was previously reported as a possible and promising marker gene for detection of *Talaromyces flavus*^20^. The replication licensing system acts to ensure that the no section of the genome is replicated more than once in a single cell cycle, while the licensing factor is protein complex that allows an origin of replication to begin DNA replication at that site^32^.

Proposed LAMP assay for *T. flavus* detection was specific and sensitive. During studies amplification was observed with DNA isolated from 5 *Talaromyces flavus* strains, while no amplification was seen with DNA isolated from 35 different fungal isolates: *Talaromyces*, *Byssochlamys*, *Neosartorya*, *Penicillium* and *Aspergillus* genera. Detection limits of developed method were: 1 fg of genomic *T. flavus* DNA, DNA isolated from soil sample contaminated with 64 *T. flavus* ascospores per one gram of sample and DNA isolated from strawberries sample contaminated with 64 *T. flavus* ascospores per one gram of sample. Moreover, proposed method remained specific in occurrence of *Byssochlamys nivea* DNA and DNA of microorganisms naturally inhabiting soil and strawberries samples. It is important to state, that proposed LAMP method turned out to be 10 times more sensitive to detection of *T. flavus* in environmental samples then the qPCR method when the same gene was used as a target for amplification^20^. Achieved limits of detection were significantly better than already published methods for detection of other fungi^42,43^.

## Conclusion

Summarizing, a sensitive and specific LAMP assay for heat resistant fungi of *T. flavus* species was developed. Proposed assay is suitable to detect referenced HRF effectively in both soil and strawberry samples. This method asserts higher sensitivity than qPCR method for detection of the same fungus, while maintain almost the same reagent cost per reaction (approximately 0.5$/per reaction)^20^, allowing to detect 1 fg of genomic *T. flavus* DNA and DNA isolated from soil or strawberry samples contaminated with 64 *T. flavus* ascospores per one gram of sample. This method contains the same drawback as many other methods based on molecular biology techniques – requires prior extraction of DNA, however very quick and simple extraction protocols, such as PrepMan Ultra Sample Preparation Reagent DNA extraction method may be employed with lower, yet satisfying detection limits of 10 fg of genomic *T. flavus* DNA and 640 ascospores per gram of soil. Moreover, thanks to potential use of different, non-instrumental detection methods such as turbidimetric, intercalating dyes or pH sensitive dyes, this method may be deployed to detect presence of *T. flavus* under field conditions. The results of this study and proposed method may be applicable and adopted for quality monitoring of *T. flavus* contamination in agriculture and food-processing industry.

## Data Availability

The datasets generated and analysed during the current study are available from the corresponding author on reasonable request.
